# Mask, Train, Repeat! Artificial Intelligence for Quantitative Wood Anatomy

**DOI:** 10.3389/fpls.2021.767400

**Published:** 2021-11-04

**Authors:** Giulia Resente, Alexander Gillert, Mario Trouillier, Alba Anadon-Rosell, Richard L. Peters, Georg von Arx, Uwe von Lukas, Martin Wilmking

**Affiliations:** ^1^Institute of Botany and Landscape Ecology, Ernst Moritz Arndt University Greifswald, Greifswald, Germany; ^2^Fraunhofer-Institut für Graphische Datenverarbeitung IGD, Rostock, Germany; ^3^CREAF, Campus de Bellaterra (UAB), Cerdanyola del Vallès, Spain; ^4^Department of Environment, Faculty of Bioscience Engineering, Ghent, Belgium; ^5^Swiss Federal Institute for Forest, Snow and Landscape Research, Birmensdorf, Switzerland; ^6^Oeschger Centre for Climate Change Research, University of Bern, Bern, Switzerland; ^7^Institute for Visual and Analytic Computing, University of Rostock, Rostock, Germany

**Keywords:** artificial intelligence, wood anatomy, deep learning, lumen area, F1 score, ROXAS

## Abstract

The recent developments in artificial intelligence have the potential to facilitate new research methods in ecology. Especially Deep Convolutional Neural Networks (DCNNs) have been shown to outperform other approaches in automatic image analyses. Here we apply a DCNN to facilitate quantitative wood anatomical (QWA) analyses, where the main challenges reside in the detection of a high number of cells, in the intrinsic variability of wood anatomical features, and in the sample quality. To properly classify and interpret features within the images, DCNNs need to undergo a training stage. We performed the training with images from transversal wood anatomical sections, together with manually created optimal outputs of the target cell areas. The target species included an example for the most common wood anatomical structures: four conifer species; a diffuse-porous species, black alder (*Alnus glutinosa* L.); a diffuse to semi-diffuse-porous species, European beech (*Fagus sylvatica* L.); and a ring-porous species, sessile oak (*Quercus petraea* Liebl.). The DCNN was created in Python with Pytorch, and relies on a Mask-RCNN architecture. The developed algorithm detects and segments cells, and provides information on the measurement accuracy. To evaluate the performance of this tool we compared our Mask-RCNN outputs with U-Net, a model architecture employed in a similar study, and with ROXAS, a program based on traditional image analysis techniques. First, we evaluated how many target cells were correctly recognized. Next, we assessed the cell measurement accuracy by evaluating the number of pixels that were correctly assigned to each target cell. Overall, the “learning process” defining artificial intelligence plays a key role in overcoming the issues that are usually manually solved in QWA analyses. Mask-RCNN is the model that better detects which are the features characterizing a target cell when these issues occur. In general, U-Net did not attain the other algorithms’ performance, while ROXAS performed best for conifers, and Mask-RCNN showed the highest accuracy in detecting target cells and segmenting lumen areas of angiosperms. Our research demonstrates that future software tools for QWA analyses would greatly benefit from using DCNNs, saving time during the analysis phase, and providing a flexible approach that allows model retraining.

## Introduction

In recent years, deep learning, as a subset of artificial intelligence, has proven to be the new key tool to investigate ecological research questions (Christin et al., 2019). Hierarchically, deep learning is a sub-field of machine learning, a modeling approach able to detect common patterns in datasets (Olden et al., 2008). The reason of deep learning suitability to ecological investigations lies in its intrinsic characteristics: deep learning can automate the pattern interpolation processes from the provided data (LeCun et al., 2015). The step forward lies in the particular algorithm architecture, which de-structures data features through different evaluation layers. This allows the machine to automatically change internal parameters and fit the computational process according to the required task (Zhang et al., 2016).

Overall, ecological investigations are enhanced by the flexibility of deep learning tools, especially when dealing with large and complex datasets (Christin et al., 2019). This is the case for image analysis tasks, where Deep Convolutional Neural Networks (DCNNs) stand out by performance (Krizhevsky et al., 2017). In this specialized architecture, the different layers are composed by artificial neurons (Zhang et al., 2016) and each layer has a specific task, such as feature extraction, mathematical computation-based training, or dimensional adjustment, that makes DCNNs particularly suitable for image interpretation (James and Bradshaw, 2020).

Wood anatomical research is a field where DCNNs find an ideal application (Garcia-Pedrero et al., 2019). In the past, machine learning methods have mainly been used for wood species identification (Luis et al., 2009; Mallik et al., 2011; Ravindran et al., 2018; He et al., 2020; Wu et al., 2021). In contrast, quantitative wood anatomy (QWA), that refers to the broad set of analyses quantifying and interpreting the variation of xylem features in trees, shrubs, and herbaceous plants (von Arx et al., 2016), has just started being investigated with such tools. Investigations are performed on wood anatomical images at a microscopic level, to study number, distribution, and properties of the main cell types: conduits, parenchyma cells, and fibers (von Arx and Carrer, 2014). Wood anatomical analyses can provide a higher temporal resolution than annual tree-ring width measurements, and the wood anatomical structure is more directly linked to biological processes and tree functioning. This allows to link tree growth to the study of phenology, tree allometry, species physiological performance, and ecosystem dynamics, among others (Fonti et al., 2010; Carrer et al., 2015; De Micco et al., 2019; Castagneri et al., 2020).

To obtain wood anatomical images, the wooden sample has to undergo several steps. Standard procedures start with cutting thin sections from the sample and mounting them on a glass slide, whereas, in case of damaged or particularly fragile material, the wood sample can be embedded in paraffin to stabilize the tissues before the cutting. This stage is followed by the image acquisition process, where images of the slides are taken with a camera installed on a microscope, or *via* specialized scanners. The images are then analyzed with image analysis tools to perform quantitative wood anatomical investigations (Gärtner et al., 2015; Yeung et al., 2015; von Arx et al., 2016; Prendin et al., 2017; Peters et al., 2018). Despite the great advances in the procedures for wood sectioning and image acquisition, the actual feature recognition phase still requires human supervision (Hwang and Sugiyama, 2021). In fact, traditional image analysis is often not able to overcome the artifacts generated by the sample processing: the great number of cells occurring in the sections, combined with a non-optimal image quality, is the reason why automated image analysis is often followed by a manual editing phase. However, the effort taken to fix these issues by hand can be very time-consuming. This is exactly where DCNNs show their strength, as many of these issues can benefit from a DCNN approach. Images showing excessive darkness, brightness or blurred areas, for example, can be easily processed after a proper neural network training. Moreover, DCNNs have the ability to encode specific wood features, which is the key to accelerate data production: fibers are less likely to be mistaken for conduits, for example, or pit chambers will be recognized as such and automatically excluded from the lumen area.

In this study, we aimed to overcome these general QWA challenges in transversal images using DCNNs. Specifically, we created a practical graphical user interface relying on a Mask-RCNN algorithm architecture (He et al., 2017), that detects cells and quantifies the lumen area on four conifers and three angiosperms. We also present the comparison on the accuracy of cell instance (cell identification as an object) and lumen area detection with another neural network architecture (U-Net; Garcia-Pedrero et al., 2019), and ROXAS, one of the most widely used programs for wood anatomical image analyses (von Arx and Dietz, 2005; von Arx and Carrer, 2014; Garcia-Pedrero et al., 2019).

## Materials

To perform classification analyses on wood anatomical parameters, such as cell instance recognition (i.e., the identification of the cell as an individual structure) and lumen area detection (cell segmentation), the neural network has to undergo a training stage. During this training, the neural network architecture is provided with wood anatomical images as well as the desired output, the ground truth – a map of the original image where all the target cells have been manually marked (segmented). Given the original and the segmented images, the algorithm can learn to generalize the features that are characteristic for a target cell. For this purpose, we chose four conifers: Norway spruce (*Picea abies* L. Karst), Scots pine (*Pinus sylvestris* L.), White spruce (*Picea glauca* Moench), and European larch (*Larix decidua* Mill); and three angiosperms: Black alder (*Alnus glutinosa* L.), European beech (*Fagus sylvatica* L.), and Sessile oak (*Quercus petraea* Liebl.). We will refer to these groups as: conifers, alder, beech, and oak. Each of them represents a typical wood anatomical structure: softwood for gymnosperms and the three typologies of hardwood for angiosperms, a diffuse-porous species, a diffuse to semi-diffuse-porous species and a ring-porous species.

Conifers, beech, and oak are some of the most used species for tree ring studies in general (Scharnweber et al., 2013; Príncipe et al., 2017; Lange et al., 2018; Janecka et al., 2020) and specifically for wood anatomical analyses (Björklund et al., 2020; Pampuch et al., 2020; Peters et al., 2020). Images were collected from several sources, to ensure variability in the image quality and within the sample processing (Supplementary Table 1). Conifer species were grouped due to the high similarity in wood anatomical structure, therefore it was possible to train one single neural network for this group; whereas the angiosperms remained separated per species, due to their less uniform structural properties. Moreover, because it is more common to perform QWA analyses on gymnosperms due to their homogeneous wood structure, having a broadly trained algorithm is of high value. Concerning gymnosperms images, the provenance is more restricted and the quality is generally very high. While for conifer images we managed to collect examples of a wide quality range, the image acquisition process of all the angiosperms slides was carried out by the high-end Zeiss Axio Scan.Z1 slide scanner (Carl Zeiss AG, Germany), except part of the alder samples.

## Methods

### Neural Network Architecture

Our goal was to perform cell instance and lumen area detection together in a single algorithm architecture to maximize the utility for QWA analyses. We treated lumen area detection as an instance segmentation problem rather than a semantic segmentation task as we see in Garcia-Pedrero et al. (2019) with the U-Net neural network architecture. The difference between these two is that with the U-Net algorithm every pixel is classified (as “cell” or “not-cell”) independently from each other, whereas in instance segmentation the objects (cells) are first identified and located as a whole, and then segmented to their estimated real dimension in a following stage. In practice, this has the benefit of fewer spurious false detections because a larger field of view can be used for the object detection stage, allowing to put distance between the image and the “observer” in order to focus better the objects themselves.

Specifically, we used the Mask-RCNN (He et al., 2017) neural network architecture for this task. This model employs a feature pyramid network (FPN) (Lin et al., 2017) to extract visual features from the input image at different levels of detail. These features are then forwarded to the next stages: first, a region proposal network (RPN) which identifies regions in the image that might be objects of interest, and second, a classification head, which filters out undesired regions. In our case this can include ray cells, pits, or air bubbles. Finally, if the region is classified as a target object, the third step performs a per-pixel segmentation. We refer to He et al. (2017) for a more detailed description of the used Mask-RCNN.

### Neural Network Training

We used the version implemented in the PyTorch v1.6 library (Paszke et al., 2019) with a ResNet50 backbone. Weights (model parameters) were obtained from pre-training on the COCO dataset (Lin et al., 2014). They were used as a starting point and then fine-tuned on image patches of anatomical wood thin sections of size 1000 × 1000 pixels for all species except oak, for which images of 2000 × 3000 pixels were used to safely include the larger vessels in the tiles. Although COCO contains natural images that have little in common with wood anatomical thin sections, this process is still beneficial as part of transfer learning. As we started algorithm development with alder, our training set consisted of 89 patches for this species, while only 31 patches were employed for conifers, 20 for beech and 20 for oak.

The images were annotated in the form of a semantic segmentation map, the ground truth, which is an image file (in png format) created for every training image, containing the information related to the correct identification of the lumen area. This map was created using Gimp (Spencer et al., 2021) a free and open-source raster graphics editor, used to draw the lumen areas on an additional layer with transparent background.

We converted this annotation into boxes by taking the minimum and maximum X and Y pixel coordinates of the connected components. During training we used data augmentation in the form of random horizontal and vertical flipping as well as 90° rotations to artificially enlarge the data set. The stochastic gradient descent (SGD) optimizer was used as the training algorithm, with a learning rate of 0.005, momentum 0.9 and weight decay of 0.0005 for overall 20 epochs and a batch size of 1.

For each wood type a separate neural network was trained. At the training stage, some modifications were required for the different species. Particularly for oak, we trained two networks that specialize in either the small or the large vessels. However, the networks belong to the same model in the graphical user interface, therefore the analyses are run in parallel, providing a single output per picture. For the large vessel network, we used patches of half the resolution (but same patch size), thus doubling the field of view.

### Neural Network Evaluation

The testing phase was performed on patches cropped from wood anatomical images of the same dimensions as the ones included in the training dataset and completely new to the algorithm (i.e., not present in the training dataset), 15 for each group. The patches were extrapolated from images of seven different trees for conifer, eight trees for alder, seven for beech, and six for oak. The aim of using this dataset was to cover all the problematic issues that might hamper a smooth workflow and require large correction efforts during the post-analysis editing phase.

Sample processing is the most delicate step in QWA analyses. When the tissue is sectioned, the fragile structure of the wooden sample can be compromised, often resulting in broken vessel or tracheid walls, and cell wall protrusion into the lumen area. The microslide preparation step is also critical as sections are very thin and can overlap with themselves, if they are not positioned carefully. Moreover, depending on the accuracy used in the following section processing, stains from coloring solutions, drops of paraffin or air bubbles can occur on the slide. If these artifacts cannot be avoided in the image acquisition step with a tailored cropping, they have to be solved manually after the automated image analysis. Acquiring high quality images can be difficult as well, since artifacts from wrongly stitched images, blurred areas due to uneven surfaces, dust spots, or inhomogeneous lighting can hamper a smooth analysis of the image and consequently, of the whole the dataset. Moreover, even the species-specific structure can hamper a traditional image analysis approach. In general, pits, fibers, apotracheal parenchyma, bark cells, vessels with scalariform perforation plates, resin canals and the related parenchyma cells constitute an issue. The testing dataset we created included at least one image for every feature mentioned above, in detail:

- Overlaying pollution particles (dirt or dust);
- Overlapping/folded tissue;
- Stains from coloring solutions of variable intensity;
- Drops of paraffin from embedding;
- Broken cell walls;
- Reduced lumen area in the latewood hampering a proper cell recognition;
- Cells that are not conduits (resin canals, bark cells, fibers, and parenchyma cells);
- Pit chambers connecting conifer tracheids;
- Pith fleck in alder;
- Scalariform perforation plate for alder and beech.

The evaluation procedure has been implemented in a web browser-based software *via* a graphical user interface, and is automated. Within the graphical user interface, four different models can be selected and applied to new images. One model corresponds to gymnosperms and one for each angiosperm. Furthermore, we implemented the possibility to upload ground truth references and previously computed prediction files. This facilitates testing and evaluating the algorithm, and also comparing its accuracy to the output of other specialized software such as the traditional image-analysis tool ROXAS (von Arx and Carrer, 2014) running on Image-Pro Plus (Media Cybernetics, 2021, Rockville, MD, United States).

For the cell instance metrics (cell detected: yes/no), the procedure first matches the connected components (cells) of the outputs with the corresponding ground truth maps. Two cells are matched if they have a high similarity (overlap) to each other and thus count as a true positive instance (TP). Unmatched cells from the output are counted as false positive instances (FP), that is when the algorithm detected a cell that is not present in the ground truth. On the other hand, unmatched cells of the ground truth are counted as false negative instances (FN), in this case cells are not present in the output and therefore they are counted as missed cells (Table 1).

**TABLE 1:** Confusion matrix approach applied to cell instance detection.

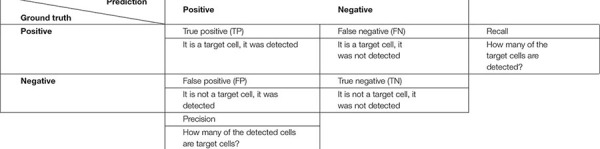

*Both recall and precision parameters are a benchmark of accuracy, respectively, they assess how many cells were missed (FN) and how many redundant cells were segmented (FP), considering the total amount of correctly detected cells (TP). See Equation 1 for reference.*

Matched target cells are then compared in terms of lumen area accuracy by directly comparing individual pixels. Pixels that are positive in both predicted and ground truth cell count as true positive lumen area. Pixels that are positive only in the predicted cell are counted as FP lumen area and those that are positive only in the ground truth cell are FN lumen area. These lumen area metrics, along with the F1 score, an index to evaluate the algorithm performance, are computed for each cell individually and results are provided as Excel files.

**FIGURE 1:**
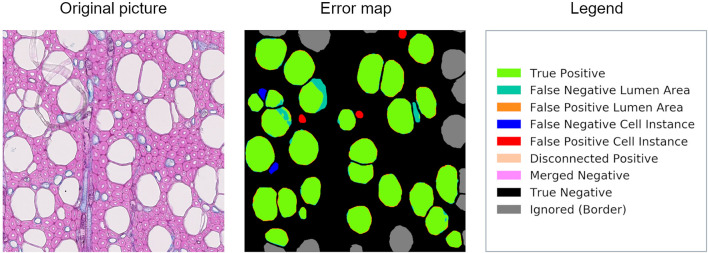
Patch from an original beech thin section, error map, and related legend. The error map is the visual comparison between the algorithm computation and the ground truth, and it helps in the visual assessment of the algorithms’ performance.

Along with the computational data, an error map is given to visually interpret the graphical user interface results on every image of the dataset (Figure 1). These image files report all the categories used to classify cell instance recognition and lumen area detection, and three other specific cases: disconnected positive, a single cell that was detected as two or more (thus counts as FP); merged negative, two or more cells that were detected as a single one (thus counts as FN); and incomplete cells, which are cut at the borders and ignored since they are irrelevant for the analysis.

**FIGURE 2:**
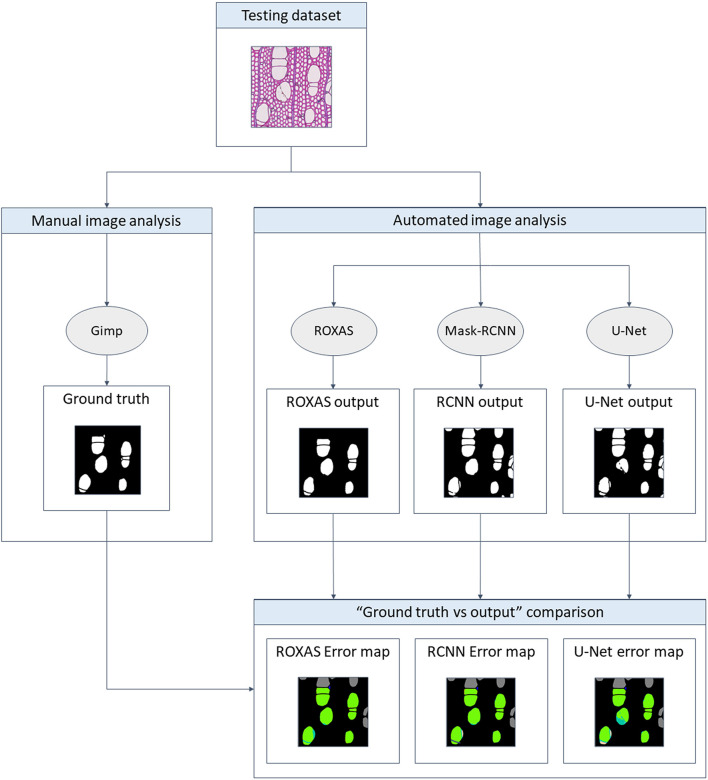
Flowchart of the comparison process. To obtain a meaningful result, the output from the algorithms has to be compared to the ground truth, a manually segmented image for every image of the groups investigated. Mask-RCNN output was then compared to the one of U-Net and ROXAS in terms of cell instance (cell detected: yes/no) and lumen area accuracy (how accurately the lumen area was detected).

The testing dataset was analyzed with ROXAS, with the Mask-RCNN algorithm, and with the U-Net algorithm. ROXAS program was run without any additional manual editing, but employing a configuration file per group species, tailored to the specific sub-datasets. Configurations are batches of settings adjusted to specific image characteristics, with the purpose of improving the performance in the cell and lumen area recognition. Subsequently, the output provided by the three approaches was compared to the ground truth, since it represents an unbiased reference (Figure 2). Results from the Mask-RCNN segmentation approach have been compared to the other two algorithms in terms of cell instance detection and lumen area detection accuracy (how accurately the cell area has been detected). Training and evaluation code can be found in the online repository: https://github.com/alexander-g/Cell-Detection-for-Wood-Anatomy.

### Cell Instance Detection and Lumen Area Detection

We defined the cells masked in the ground truth as target cells because they represent the optimal image analysis output, in this case, they are represented by vessels for angiosperms and tracheids for gymnosperms. As a first step, we evaluated how many target cells were correctly identified; furthermore, we analyzed and compared the results with the other two algorithms: ROXAS and U-Net. The latter is the same architecture used in Garcia-Pedrero et al. (2019). In this study, U-Net was employed with the same purpose but trained on ROXAS output as ground truth, while in our research the ground truth was manually created, allowing first, a consistent training of the neural network models, and second an unbiased reference for comparing the algorithms. For the same purpose, in our study, the U-Net neural network architecture was trained on the same training dataset as the Mask-RCNN version. Comparing U-Net performance with Mask-RCNN should allow us to provide a more complete overview on the topic and to highlight the improvements in this research field.

Cell instance accuracy was assessed with the help of a confusion matrix (Table 1). The confusion matrix helps to bring together and compare the results from the two sides: the algorithm (Mask-RCNN, ROXAS, or U-Net) and the ideal output (ground truth).

False positive and false negative values were used to analyze recall, which estimates the missed cells numbers (FN) considering the total TP detected; and precision, which gives an estimation of the redundant cells (FP) weighting the value with TP (Equation 1, 2).


(1) $$R\,e\,c\,a\,l\,l=\frac{T\,P}{T\,P+F\,N}$$

(2) $$P\,r\,e\,c\,i\,s\,i\,o\,n=\frac{T\,P}{T\,P+F\,P}$$

A general overview of the performance of the algorithm in cell instance detection is provided by the F1 score. This index is the harmonic mean between precision and recall (Equation 3), for this reason it reflects both when the cell instance count is overestimated and/or when it is underestimated.


(3) $$F_{1}=2*\frac{P\,r\,e\,c\,i\,s\,i\,o\,n*R\,e\,c\,a\,l\,l}{P\,r\,e\,c\,i\,s\,i\,o\,n+R\,e\,c\,a\,l\,l}$$

Computing TN for the cell instance analysis, does not apply in this case, since there is no reference on the number of total cells of all the typologies (vessels/tracheids, fibers, rays, and axial parenchyma) present in the images. The definite and important value is the number of target cells shown by the patches, obtained *via* the ground truth mask. Overall, we calculated the confusion matrix for all the species and for all the segmenting approaches (Supplementary Table 2).

After assessing how many cells were correctly identified, we analogously (i.e., with the same recall, precision, and F1 score scheme) evaluated the accuracy of the cell lumen area detection, in the following referred to as lumen area. In fact, parameters belonging to the cell instance classification can be applied to the pixel dimension as well: pixels correctly assigned to the cell area are TP and those which are misclassified belong to the FP or FN categories. We used the F1 score to provide an overall assessment of the algorithms’ performance, and subsequently analyzed precision and recall to understand when the lumen area is overestimated and/or when it is underestimated.

Moreover, an additional sub-dataset was created to increase variability of the original testing dataset, and to test the ability of the Mask-RCNN algorithm to handle the most insidious issues faced in QWA analyses (see list in section “Neural Network Evaluation”). This dataset consisted of five images per group (conifer, alder, beech, and oak) that were not analyzed with ROXAS, since they would have required a tailor-made configuration, which requires expert knowledge.

## Results and Discussion

### Result Structure

**FIGURE 3:**
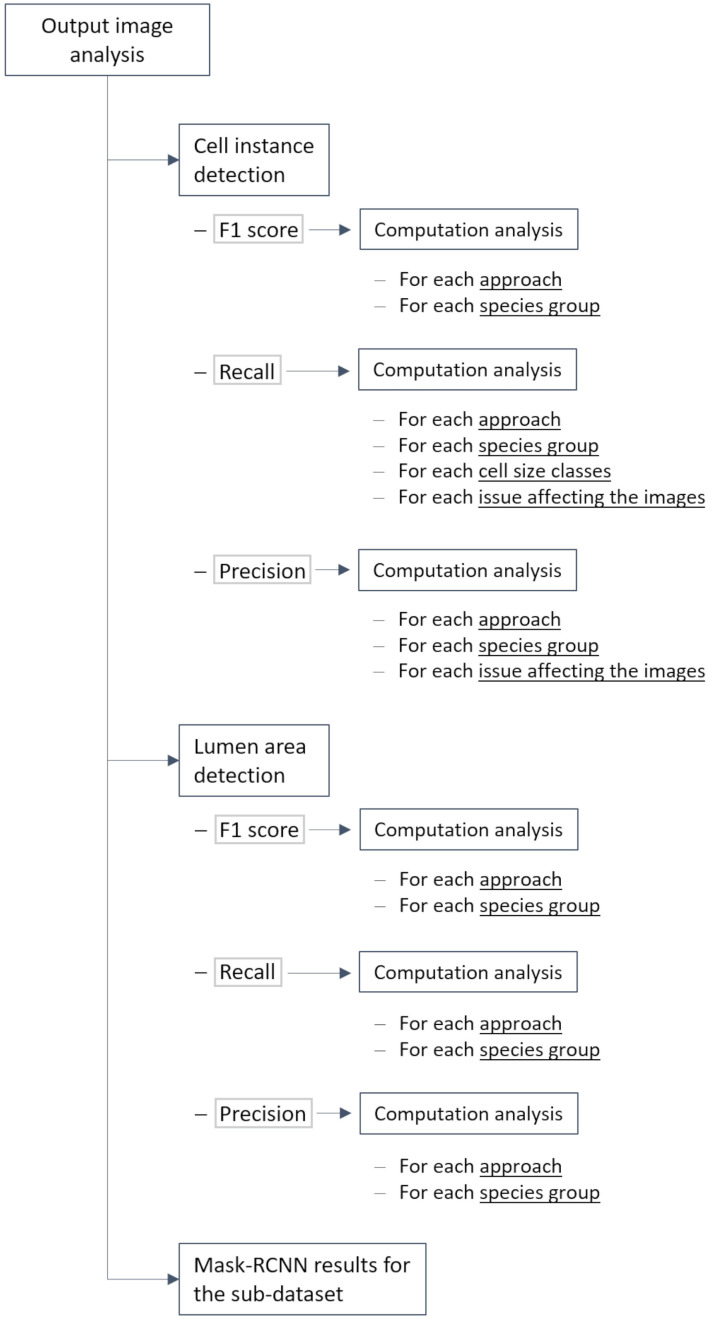
Flowchart summarizing how performance of the considered approaches was assessed.

The relatively complex analysis structure used to highlight different aspects of algorithm performance as described in section “Methods” is summarized in Figure 3 for convenience.

### Cell Instance Detection

First, performances on cell instance detection have been calculated using the F1 score (Table 2). Since the values provide an overall assessment of the accuracy reached by the algorithms, we could already infer that in general Mask-RCNN and ROXAS records were very high and that these two approaches performed very similarly for all the groups, while U-Net consistently showed lower values.

**TABLE 2:** Average values of F1 score, recall, and precision for cell instance detection using different segmenting approaches (Mask-RCNN, U-Net, and ROXAS) calculated for all anatomical groups.

	Conifer			Alder			Beech			Oak		
				
	Mask-RCNN	U-Net	ROXAS	Mask-RCNN	U-Net	ROXAS	Mask-RCNN	U-Net	ROXAS	Mask-RCNN	U-Net	ROXAS
F1	0.93	0.91	0.94	0.88	0.83	0.88	0.92	0.80	0.92	0.93	0.85	0.89
Recall	0.97	0.99	0.97	0.96	0.96	0.92	0.91	0.95	0.89	0.93	0.92	0.85
Precision	0.89	0.84	0.91	0.82	0.73	0.84	0.94	0.69	0.96	0.94	0.79	0.94

As the F1 score is the harmonic mean between precision and recall, it raised interest in the investigation of the algorithms’ trend about underestimation and overestimation of the cell instance detection. The recall parameter was generally higher for the U-Net algorithm, followed by Mask-RCNN and ROXAS (Table 2). High recall values indicate that, overall, fewer cells are missed compared to the total amount of cells correctly recognized. Although this might seem in contrast to the F1 score results, the U-Net approach of recognizing cells – pixel per pixel – seems to lose the perspective on the cell instance identification, generating many spurious cells detection, therefore rising the probability of segmenting the right ones. Conifer was the group showing the best result for all algorithms (0.99 for U-Net, 0.97 for Mask-RCNN, and ROXAS); while alder, beech, and oak vessels segmentation showed very similar results for the artificial intelligence algorithms (0.96, 0.91, and 0.93 for Mask RCNN, and 0.96, 0.95, and 0.92 for U-Net, respectively). Slightly lower values were shown for ROXAS, where oak was the species with the lowest recall value (0.85).

**FIGURE 4:**
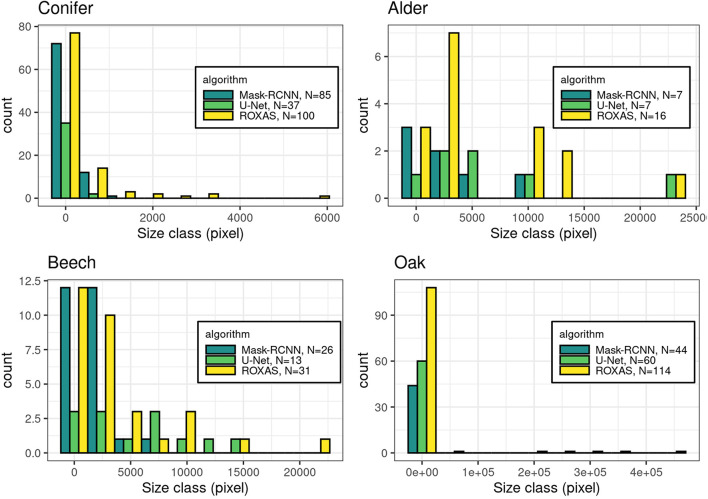
Histograms of the missed cells (FN value) per groups: conifer, alder, beech, and oak. The count in the legend refers to the FN instances that were recorded in total per algorithm for each species, while the histograms show the respective size-frequency distributions.

Furthermore, we investigated the characteristics of the FN instance within the cell size classes, to determine where the lack of cell detection was more pronounced (Figure 4). This assessment is useful for the future training of the DCNN, in order to fine-tune the cell instance detection process. Generally, the most affected size classes are the smaller ones, but wood anatomical type plays a consistent role in this evaluation. Oak as a ring-porous species shows great differences in size between the earlywood the latewood vessels. This size difference exists also in conifers, where latewood cells are distinctively smaller than the earlywood cells. However, in conifers the difference is smaller and the transition occurs less abruptly. In these two categories, the most affected size classes were the smallest, supporting the predictable trend that the smallest cells are more difficult to identify. In contrast, alder and beech, diffuse and semi-diffuse porous species, respectively, show the bias shifted to medium-small cells and more evenly distributed across size classes. This analysis demonstrates that, when the wood structure is more homogeneous, cell size has less effect on cell instance recognition.

**FIGURE 5:**
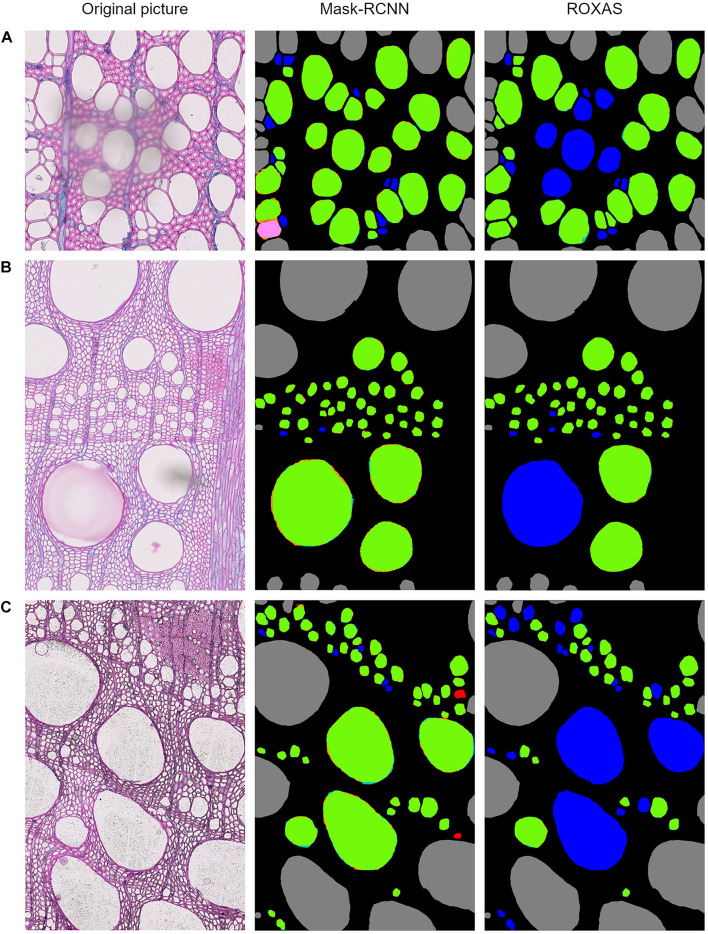
Output comparison between Mask-RCNN and ROXAS on examples of overlaying dust particle **(A)**, paraffin drops **(B),** and stains of coloring solutions **(C)**. Error legend can be found in Figure 1. The FN cell instance are marked in blue, and in ROXAS outputs they exactly correspond to the area interested by the **(A–C)** issue.

Since the testing dataset was built to include artifacts generated by sample preparation, we analyzed how the detection process is affected when an artifact occurs, comparing the Mask-RCNN segmentation approach with ROXAS. As a result, we observed that tackling FN issues from the segmentation methodology perspective highlights the benefit of using artificial intelligence for the feature recognition step. A visual comparison between Mask-RCNN output and the ROXAS output, involving the issues that might generate FN instances, is shown in Figure 5: blurred areas caused by various artifacts such as overlapping object (Figure 5A), paraffin drops (Figure 5B), and stains of coloring solutions (Figure 5C).

To estimate how many non-target (redundant) cells got recognized, we used the precision parameter: the closer to 1 the value, the lower is the overestimation in cell instance detection.

As we expected, U-Net architecture provided the lowest values, therefore the worst performance. Mask-RCNN and ROXAS results showed the same general pattern: from the worst to the best precision rate we found alder (0.82, 0.84), conifers (0.89, 0.91), oak (0.94 for both), and beech (0.94, 0.96). Overall, results on the precision values were very high and close for both approaches, although ROXAS demonstrated to be slightly more efficient in the task (Table 2).

Precision values interpretation was performed on a visual level, comparing the error maps provided by the different algorithms, to identify in which situations errors occurred more frequently and which cells were most susceptible. Since the U-Net architecture proved not to be particularly meaningful in filtering target cells from non-target cells, we focused on the comparison between ROXAS and Mask-RCNN outputs (Supplementary Table 3).

The wood anatomical feature that produced the most FP instances was the bark, with similar results for both approaches. In this respect, ROXAS provides the possibility to define an area of interest and thus to manually exclude the bark. Nevertheless, the comparison was done on images analyzed without any manual editing (no tailored area of interest defined), and it was performed with the same methodology than with the Mask-RCNN algorithm. The process could be further streamlined if the algorithm training focused on bark/non-bark area recognition, since the manual editing step of creating the area of interest could be skipped.

Overall, the cell categories presenting FP issues coincide for the Mask-RCNN algorithm and ROXAS on conifers. However, one advantage of the Mask-RCNN seems to be pit recognition. The high amount of target cells per unit area allowed the algorithm to perfectly recognize this feature, and no pit was included in the lumen area nor mistaken for a cell itself for every image of the dataset analyzed.

**FIGURE 6:**
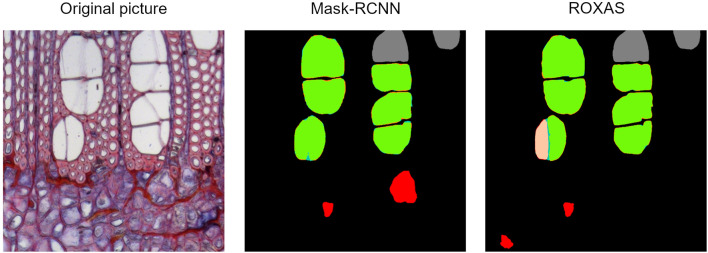
Output comparison on vessel identification. Original alder image from the testing dataset, Mask-RCNN error map, and ROXAS error map. When the perforation plate is strongly visible, there is a high chance that one single vessel is wrongly recognized as two separate ones.

For the angiosperms, Mask-RCNN FPs included all those small cells that could resemble a small vessel (our target), or a big fiber or apotracheal parenchyma; while ROXAS automatically filtered them out by their size thanks to the tailored configuration files. Nonetheless, we believe that this challenging category cannot be clarified unambiguously without a closer look at the longitudinal sections of a sample. This highlights how the learning process of the DCNN approach really took place, and clearly had an effect on the image segmentation. Another example of FP included the scalariform perforation plates (Figure 6), a feature abundantly present in alder and occasionally in beech. Here the algorithm, within certain limits, successfully distinguished between adjacent vessels and individual vessels sectioned at a scalariform perforation plate with thin bars, which could be wrongly interpreted as two adjacent vessels. The interpretation as one or two separate conduits influences the calculation of theoretical hydraulic conductivity of the specimen and therefore matters for studies dealing with water transport.

The Mask-RCNN output presented some FP instances related to damaged cells. This happened because the algorithm training aimed to consider every cell whose shape can be accurately predicted, as long as it fulfills the requirements for a reliable measurement. What is usually a matter of exclusion when the whole dataset is of average quality, sometimes has to be reconsidered as usable if this happens to be the only material available, therefore as many cells as possible should be measured. In this testing dataset, the slide quality was very high and quite homogeneous, which explains why many high-quality cells were available and thus damaged and dubious cells were not segmented in the ground truth. This sheds light on how FP evaluation is very case-specific regarding this issue. However, applied to QWA analyses and specifically to ROXAS use, the FP error can be considered less time-consuming to handle, since eventually requires deleting redundant cells, rather than drawing new ones from scratch, as it happens in the case of FNs.

### Lumen Area Detection

Overall, all segmentation approaches showed very high F1 scores for lumen area (Table 3). For conifers, values varied between 0.98 (ROXAS) to 0.95 (Mask-RCNN algorithm). A different trend characterized the angiosperms. The Mask-RCNN algorithm results for alder, beech, and, oak, all reported an F1 score of 0.97. This value was the same for ROXAS with beech and oak, but it was slightly lower (0.96) for the ROXAS segmentation results on alder. U-Net algorithm did not cope as well as the other approaches, and while the value was still very similar for alder (0.95), it dropped for beech (0.93), and oak (0.91).

**TABLE 3:** Average values of F1 score, recall, and precision for lumen area detection using different segmenting approaches (Mask-RCNN, U-Net, and ROXAS) calculated for all the groups.

	Conifer			Alder			Beech			Oak		
				
	Mask-RCNN	U-Net	ROXAS	Mask-RCNN	U-Net	ROXAS	Mask-RCNN	U-Net	ROXAS	Mask-RCNN	U-Net	ROXAS
F1	0.95	0.96	0.98	0.97	0.95	0.96	0.97	0.93	0.97	0.97	0.91	0.97
Recall	0.97	0.94	0.97	0.98	0.94	0.94	0.97	0.92	0.96	0.97	0.90	0.97
Precision	0.93	0.98	0.98	0.96	0.98	0.97	0.97	0.97	0.99	0.97	0.96	0.98

**FIGURE 7:**
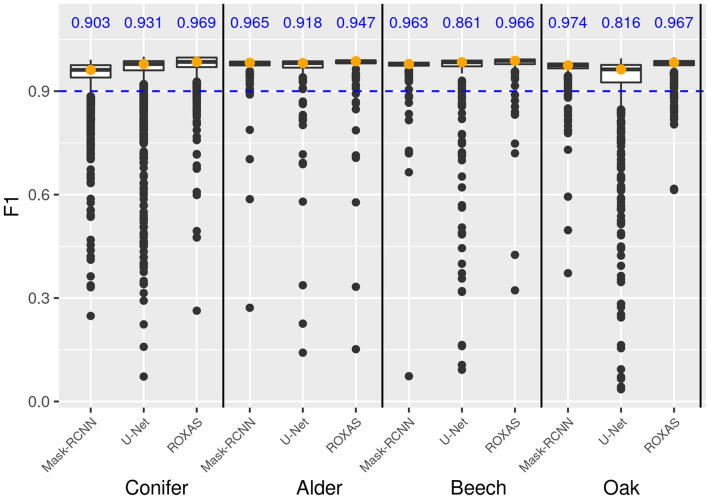
Box plot showing the frequency distribution of the F1 score for lumen area, which allows a comparison of the three algorithms (Mask-RCNN, U-Net, and ROXAS) for each tree-species group. The blue values represent the fraction of cells reaching and surpassing the F1 score threshold of 0.9.

Overall, F1 scores were very high and results were close for all the segmenting approaches, but generally ROXAS performed slightly better. Nonetheless, since with artificial intelligence we aimed at very high accuracy levels to avoid manual editing as much as possible, we additionally analyzed the percentage of cells belonging to the highest accuracy class, that is when F1 ≥ 0.9 (Figure 7, blue number). U-Net underperforms for beech and oak and to a lesser extent for alder. The Mask-RCNN algorithm and ROXAS perform generally similarly well for the angiosperms, but Mask-RCNN performs lower in the conifer category.

**FIGURE 8:**
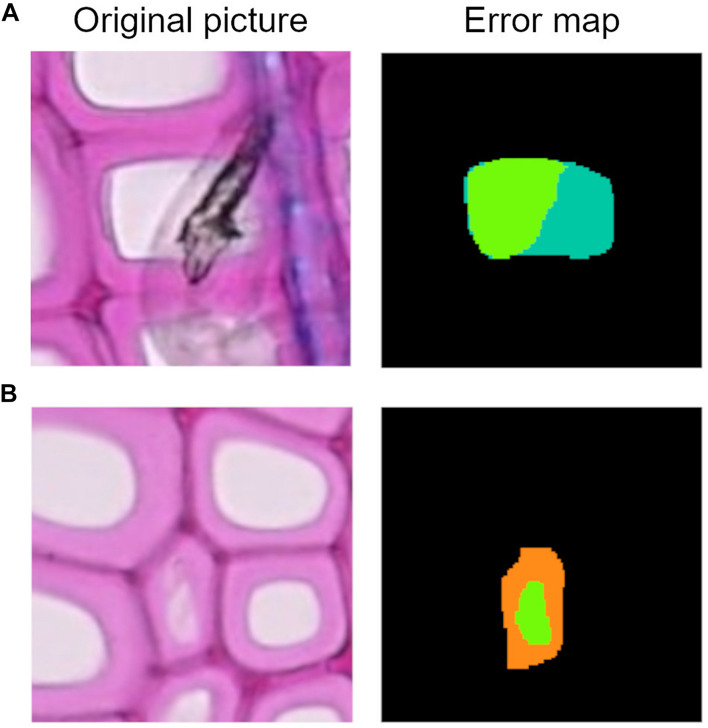
**(A)** Example of lumen area underestimation, the dark green area represents the FN pixels. **(B)** Example of lumen area overestimation, the orange area represents the FP pixels.

The F1 score gives an overall idea of how well lumen area is detected. To understand if the algorithms are generally underestimating or overestimating the areas, we need to closely analyze precision and recall parameters. In Table 4 we show two cell records from the conifer dataset generated by the Mask-RCNN algorithm. Both show very low F1 scores, meaning that the segmentation process failed in accuracy for both cells. In the first row, it is shown how precision, which ranges from 0 to 1, reaches its maximum, while recall is very low. In practice, what happened is that all the pixels that were recognized as belonging to the cell were indeed target pixels, but not all the target pixels were recognized, leading to an underestimation of the area (Figure 8A). The second row in Table 4 reports the opposite situation, recall reaches the maximum value, but precision is low. Figure 8B visually explains this case: all the pixels belonging to the cell were recognized, but redundant pixels around the area were included in the segmentation, resulting in an overestimated lumen area.

**TABLE 4:** Example of parameters calculated for an underestimated lumen area and an overestimated lumen area.

	Area	Prediction	TP	FP	FN	Precision	Recall	F1
Underestimated lumen area	1715	482	482	0	1233	1	0.28	0.44
Overestimated lumen area	85	600	85	515	0	0.14	1	0.25

*When high precision is associated to low recall, then the area is underestimated. When the values are low for precision and high for recall, then the area is overestimated.*

Results for all the three approaches demonstrated a very high performance, even in terms of precision and recall of lumen area segmentation (Table 3). In the conifer group, we found that ROXAS had the highest F1, precision, and recall values (0.98, 0.98, 0.97, respectively). This resulted in very few redundant pixels and even less missed ones. Regarding the deep learning approaches, cells were more likely to be underestimated with U-Net (0.94 for recall and 0.98 for precision), while the opposite held for Mask-RCNN (0.97 for recall and 0.93 for precision). Analyses of alder samples were most precise when performed by the Mask-RCNN algorithm. The advantage of this approach consists of a lower percentage of lumen area underestimated (0.98 for recall), and still a very good result on the overestimation issue (0.96 for precision). ROXAS and U-Net, in contrast, tend to underestimate cell areas (0.94 for both approaches on recall) more than overestimating them (0.97 for ROXAS and 0.98 for U-Net on precision). Beech and oak segmentation behaved very similarly in the three approaches. Mask-RCNN was the most stable of the three, where 0.97 was the value for both species and for both parameters, meaning that there was no evident tendency in under- or over-estimating. ROXAS, which performs better than U-Net, showed a strong precision both on beech (0.99) and oak (0.98), but generally underestimated lumen area. The same trend was confirmed for U-Net, with a stronger tendency in underestimating lumen area (0.92 for beech and 0.90 for oak).

Since many aspects contribute to the evaluation process of the algorithms’ performances, we include a general overview of the results to summarize the main outcomes of this analysis (Table 5).

**TABLE 5:** Summary result on the comparison of the algorithms’ performances.

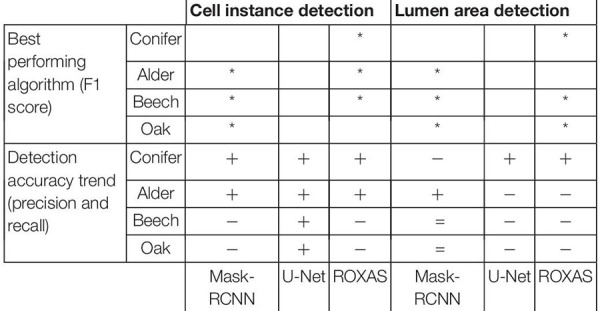

The best performing algorithm was signaled with an asterisk (*). If the F1 score of a group type reported the same number for more than one approach, the star is represented twice. With respect to the detection accuracy trend, the overestimation both of the cell instance and the lumen area was reported with a plus sign (+), underestimation with a minus (−), and if there was no evident trend an equal symbol was noted (=).

### Mask-RCNN Results for the Sub-Dataset

The Mask-RCNN approach worked properly for all the species and accurately segmented cells (Supplementary Figure 1). The visual assessment revealed that conifers remain the main weakness of the Mask-RCNN. The most occurring issue is represented by missed cells, especially small cells in the latewood. Regarding the lumen area detection, underestimation was more likely to happen, but the algorithm coped particularly well with problematic regions where the cell wall protrudes in the lumen area or where stains occurred. Alder is one of the species the model worked best with: error maps showed few redundant vessels and some broken ones wrongly segmented. This great performance is partially explained by the fact that the neural network could rely on a larger training dataset, but most likely by the fact that vessels have a distinctive shape and size compared to the surrounding fibers, and the diffuse porous organization of the vessels make them quite consistent in shape along the ring. If the larger training dataset would have had a major impact, we would have also noticed a difference within the groups in the F1 scores calculated for the main dataset. In general, for alder images, a very small portion of target cells were missed, in contrast to beech. Beech presented very accurately segmented lumen areas but some of them, especially when affected by very dark stains, were not identified. The large oak earlywood vessels were almost perfectly recognized, with only a minor percentage of missing cells and some redundant fibers which were segmented in the latewood.

In this smaller sample we also tested the running time of the Mask-RCNN model, using a 15-10210U CPU. By monitoring the process with a timer, we found out that the time needed for analyzing cells per single image varied from a minimum of 7.76 s to a maximum of 1.37 min depending on the species and the complexity of the image. Running time is also dependent on the task; when the comparison between other outputs is performed along with the cell detection, time can slightly increase. On average, oak is the species that employs more time because of the two models running on the same image.

## Conclusion

Our results show that Mask-RCNN is highly suitable for the analysis of wood anatomical images. In all four wood-type groups, cells could be detected and segmented with high accuracy (i.e., high F1 score, precision, and recall).

While ROXAS always performed better for conifers in all the different parameters analyzed, the Mask-RCNN was better suited for angiosperms. We can thus infer that the wood anatomical variability of the angiosperms does not hamper a proper segmentation process with the Mask-RCNN algorithm. This is explained by the methodology employed by the Mask-RCNN approach: the instance segmentation operated by the algorithm first evaluates the context of a target cell and then proceeds in the segmentation. Therefore, the more stable the features of the target cells in the images, the more likely the Mask-RCNN is to succeed in the detection process, despite the diversity of the surrounding structures. Oak vessels, for example required two different algorithms, due to the difference in shape and size. Analogously, the homogeneous pattern of conifers is not facilitating a proper detection and segmentation of the tracheids, due to the small lumen of the latewood cells.

Another important aspect is the perspective we take to look at the data. A strict evaluation of numeric results would suggest that Mask-RCNN accomplishes the best performance for the F1 score with three species categories out of four; U-Net performed best for the recall parameter, very close to the Mask-RCNN results; and ROXAS recorded the best values for the precision parameter, still very close to Mask-RCNN results (Table 2). However, the visual interpretation of the error maps allowed us to draw additional conclusions. Looking at the recall value on cell instances (i.e., missed cells) from the cell characteristic perspective, highlighted how the detection process benefits from the neural network methodology. Many of the issues we selected, that hamper a smooth workflow with a traditional image analysis approach (i.e., ROXAS), were better handled with the DCNNs. Both U-Net and Mask-RCNN showed not only the best results, but also a similar trend. At the same time, visual interpretation of the error maps was also important for the analysis of redundant cell instances (i.e., precision). If on a first look, ROXAS and Mask-RCNN seemed to behave similarly, a further analysis demonstrated the Mask-RCNN ability to encode species-specific features, thus avoiding certain undesirable cell categories that we previously classified as issues. Moving the focus to the precision of the lumen area detection and the fraction of target cells reaching and surpassing a 0.9 threshold for the F1 score, we obtained the highest values for Mask-RCNN in all the angiosperm groups. Although Mask-RCNN was not always the best performing, it demonstrated to be the most stable concerning the underestimation and overestimation of lumen area parameter. Overall, the most frequent issue hampering the lumen area segmentation was the underestimation of lumen areas.

As expected, the Mask-RCNN is best suited for the detection of a high numbers of objects in a single image. As previously stated, the algorithm first detects all the possible target cells, therefore a consistent number of redundant predictions are avoided. Because segmenting a higher number of cells makes it more likely to identify all the target cell instances, recall values showed the highest results for U-Net model.

Overall, ROXAS performed very well, despite the traditional image analysis methodology. This is partially explained by the characteristics of the testing dataset. The selected images already belonged to a very high-quality standard, i.e., they were quite homogeneous and tissue identification was generally very clear. Moreover, ROXAS configuration files used to analyze the images were specifically created for each dataset, to obtain the best performance from the program. In contrast, we demonstrated how the Mask-RCNN model can deal with bad quality samples reaching an acceptable result, even with a rather small training dataset (at least 30 images per group), consisting of cropped images from original sections. The development of a very flexible and user-friendly tool is particularly beneficial for future studies on various species. For this reason, our next aim is to implement the current version with the ability of retraining the models as a permanent feature. Generally, the advantage of the Mask-RCNN approach does not rely in a high-speed processing of the images, but if the performance in detecting and segmenting target cell is high, then the manual editing phase afterward can be avoided or significantly reduced.

In summary, this study shows that future QWA analyses could greatly benefit from Mask-RCNN approaches, such as the one presented here, due to their high accuracy, stability, and ability to deal with artifacts, coupled with high usability. Moreover, a highly automatized approach, like Mask-RCNN, will allow the processing of larger quantities of wood anatomical measurements in a shorter time, opening the way for higher replicated studies on variability in wood anatomical features.

## Data Availability Statement

The raw data supporting the conclusions of this article will be made available by the authors, without undue reservation.

## Author Contributions

GR, MT, and MW designed the study with the contribution of AA-R. AG and UL provided the user interface and the models for the algorithm. GR, GA, AA-R, RP, and MT contributed with data. GR and MT performed the measurements and statistic assessment. GR wrote the manuscript with the contribution of all the co-authors. All authors contributed to the article and approved the submitted version.

## Conflict of Interest

The authors declare that the research was conducted in the absence of any commercial or financial relationships that could be construed as a potential conflict of interest. The reviewer AB declared a past collaboration with one of the authors MW to the handling editor.

## Publisher’s Note

All claims expressed in this article are solely those of the authors and do not necessarily represent those of their affiliated organizations, or those of the publisher, the editors and the reviewers. Any product that may be evaluated in this article, or claim that may be made by its manufacturer, is not guaranteed or endorsed by the publisher.
