# Supplementary material for: Mask, Train, Repeat! Artificial Intelligence for Quantitative Wood Anatomy
Source: Front Plant Sci. 2021 Nov 4;12:767400. doi: 10.3389/fpls.2021.767400 (PMC8601631; doi:10.3389/fpls.2021.767400)
Supplement: Supplementary file 1 [file Data_Sheet_1.docx]

Table S1. List of the species and the related additional information: sample processing, image acquisition technique, and source.

| Species | Sample processing | Image acquisition technique | Source |
| --- | --- | --- | --- |
| *Alnus glutinosa* L. | Common slide protocol | - Leica DFC450C, Leica DM2500 (camera + microscope system) | AA-R |
| *Picea abies* L. | Common slide protocol | - D-Sight 2.0 (slide scanner)  - Leica DFC450C, Leica DM2500 (camera + microscope system) | Angela Luisa Prendin |
| *Picea abies* L.;  *Pinus sylvestris* L.; *Fagus sylvatica* L. | Paraffin embedding | Zeiss Axio Scan.Z1 | GvA |
| *Alnus glutinosa* L., *Pinus sylvestris* L.; *Fagus sylvatica* L. | Common slide protocol for *Fagus* and *Alnus*,  Paraffin embedding for *Pinus* | Zeiss Axio Scan.Z1 | GR |
| *Picea glauca* Moench; *Pinus sylvestris* L. | Common slide protocol | - D-Sight 2.0 (slide scanner)  - Leica DFC450C, Leica DM2500 (camera + microscope system) | (Lange et al. 2020) |
| *Picea glauca* Moench | Common slide protocol | - Leica DFC450C, Leica DM2500 (camera + microscope system) | MT |
| *Larix decidua* Mill; *Pinus sylvestris* L.; *Fagus sylvatica* L.; *Quercus petraea* Liebl. | Paraffin embedding | Zeiss Axio Scan.Z1 (slide scanner) | RLP |
| *Picea glauca* Moench | Common slide protocol | Zeiss Axio Scan.Z1 (slide scanner) | (Pampuch et al. 2020) |

Table S2. Computation of TP, FP, FN, precision and recall for all the species groups included in the dataset: conifer, alder, beech, and oak.

Conifer

| Prediction  Ground  truth | Positive | | | | | Negative | | |  | | |
| --- | --- | --- | --- | --- | --- | --- | --- | --- | --- | --- | --- |
| Positive | True Positive | | | | | False Negative | | | Recall | | |
|  | RCNN | U-Net | | ROXAS | | RCNN | U-Net | ROXAS | RCNN | U-Net | ROXAS |
|  | 2923 | 2944 | | 2908 | | 85 | 37 | 100 | 0.97 | 0.99 | 0.97 |
| Negative | False Positive | | | | | True Negative | | |  | | |
|  | 293 | | 573 | | 297 | -- | | |  |  |  |
|  | Precision | | | | |  | | |  |  |  |
|  | 0.91 | | 0.84 | | 0.91 |  |  |  |  |  |  |

Alder

| Prediction  Ground  truth | Positive | | | | | Negative | | |  | | |
| --- | --- | --- | --- | --- | --- | --- | --- | --- | --- | --- | --- |
| Positive | True Positive | | | | | False Negative | | | Recall | | |
|  | RCNN | U-Net | | ROXAS | | RCNN | U-Net | ROXAS | RCNN | U-Net | ROXAS |
|  | 198 | 195 | | 188 | | 7 | 7 | 16 | 0.96 | 0.96 | 0.92 |
| Negative | False Positive | | | | | True Negative | | |  | | |
|  | 32 | | 73 | | 35 | -- | | |  |  |  |
|  | Precision | | | | |  | | |  |  |  |
|  | 0.86 | | 0.73 | | 0.84 |  |  |  |  |  |  |

Beech

| Prediction  Ground  truth | Positive | | | | | Negative | | |  | | |
| --- | --- | --- | --- | --- | --- | --- | --- | --- | --- | --- | --- |
| Positive | True Positive | | | | | False Negative | | | Recall | | |
|  | RCNN | U-Net | | ROXAS | | RCNN | U-Net | ROXAS | RCNN | U-Net | ROXAS |
|  | 269 | 280 | | 264 | | 26 | 13 | 31 | 0.91 | 0.95 | 0.89 |
| Negative | False Positive | | | | | True Negative | | |  | | |
|  | 15 | | 124 | | 9 | -- | | |  |  |  |
|  | Precision | | | | |  | | |  |  |  |
|  | 0.95 | | 0.69 | | 0.97 |  |  |  |  |  |  |

Oak

| Prediction  Ground  truth | Positive | | | | | Negative | | |  | | |
| --- | --- | --- | --- | --- | --- | --- | --- | --- | --- | --- | --- |
| Positive | True Positive | | | | | False Negative | | | Recall | | |
|  | RCNN | U-Net | | ROXAS | | RCNN | U-Net | ROXAS | RCNN | U-Net | ROXAS |
|  | 741 | 717 | | 671 | | 44 | 60 | 114 | 0.94 | 0.92 | 0.85 |
| Negative | False Positive | | | | | True Negative | | |  | | |
|  | 52 | | 191 | | 46 | -- | | |  |  |  |
|  | Precision | | | | |  | | |  |  |  |
|  | 0.93 | | 0.79 | | 0.94 |  |  |  |  |  |  |

Table S3. False Positive (FP) count analyzed for each cell category. When the algorithm, Mask-RCNN or ROXAS, produces false positive instances related to the specific cell category, then a plus sign (+) is assigned, highlighted also by the darker background; otherwise a minus (-) is noted.

| Conifers | Pit | Ray | Resin canal | Parenchyma cells around resin canal | Artifacts cell from folded thin sections | Broken cell (to be avoided) | Intercellular spacing in compression wood | Bark cell |
| --- | --- | --- | --- | --- | --- | --- | --- | --- |
| Mask-RCNN | - | **+** | **+** | **+** | **+** | **+** | **+** | **+** |
| ROXAS | **+** | **+** | - | **+** | **+** | **+** | **+** | **+** |

| Angiosperms | Algorithm | Fiber | Bark cell | Pith fleck | Rays | Broken cell  (to be avoided) | Axial parenchyma | Vasicentric tracheid | Apotracheal parenchyma |
| --- | --- | --- | --- | --- | --- | --- | --- | --- | --- |
| Alder | Mask-RCNN | **+** | **+** | **+** | - | **+** | - | - | - |
|  | ROXAS | **+** | **+** | **+** | - | - | - | - | - |
| Beech | Mask-RCNN | **+** | **+** | **-** | - | **-** | **+** | **-** | **-** |
|  | ROXAS | **-** | **+** | **-** | - | **-** | **+** | **-** | **-** |
| Oak | Mask-RCNN | **-** | **+** | **-** | - | **+** | **-** | **+** | **+** |
|  | ROXAS | **-** | **+** | **-** | **+** | **-** | **-** | **+** | **+** |


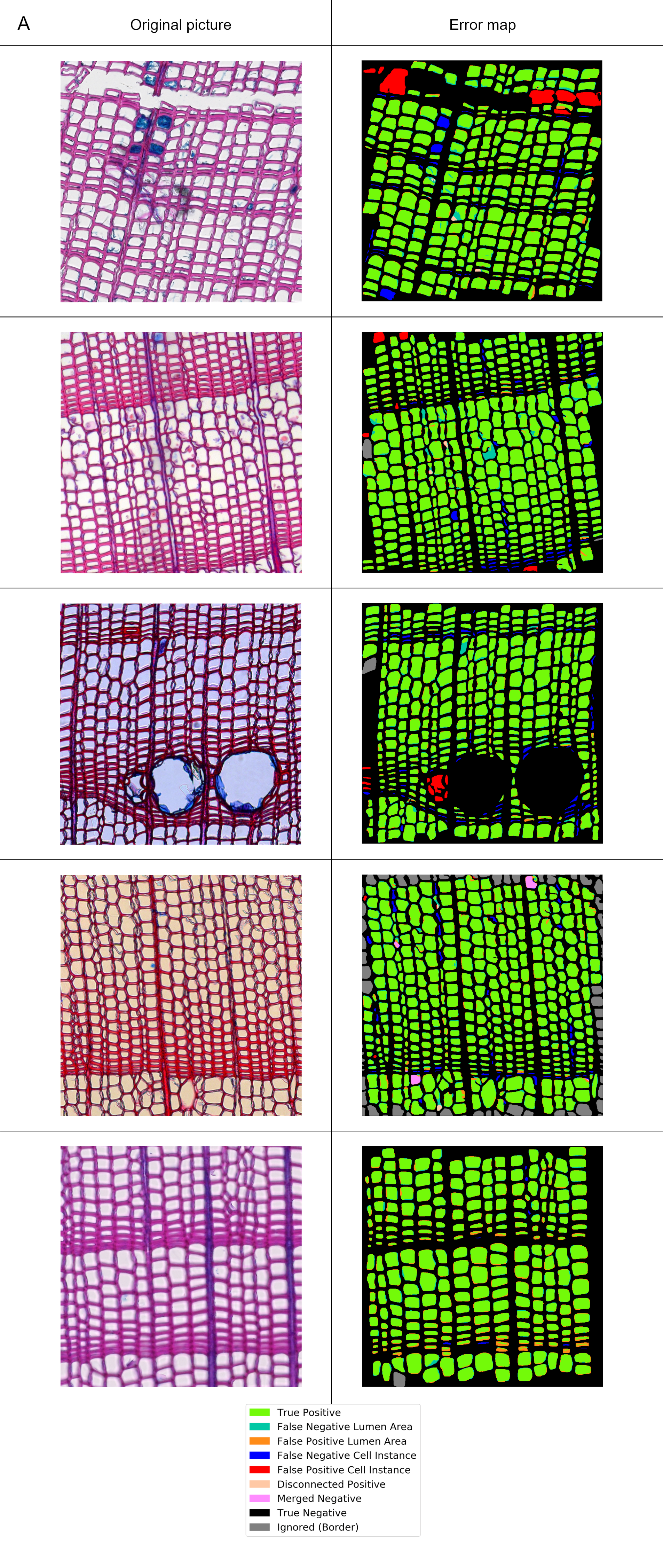

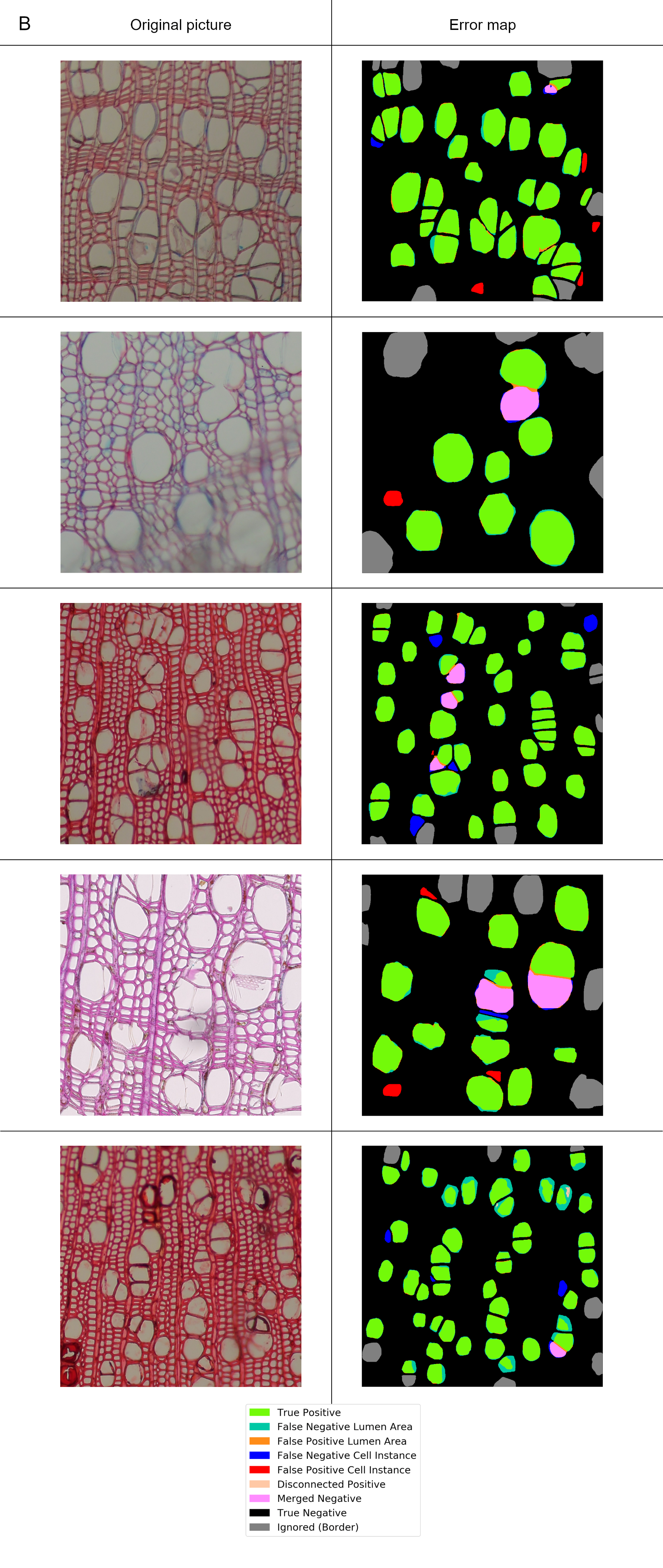


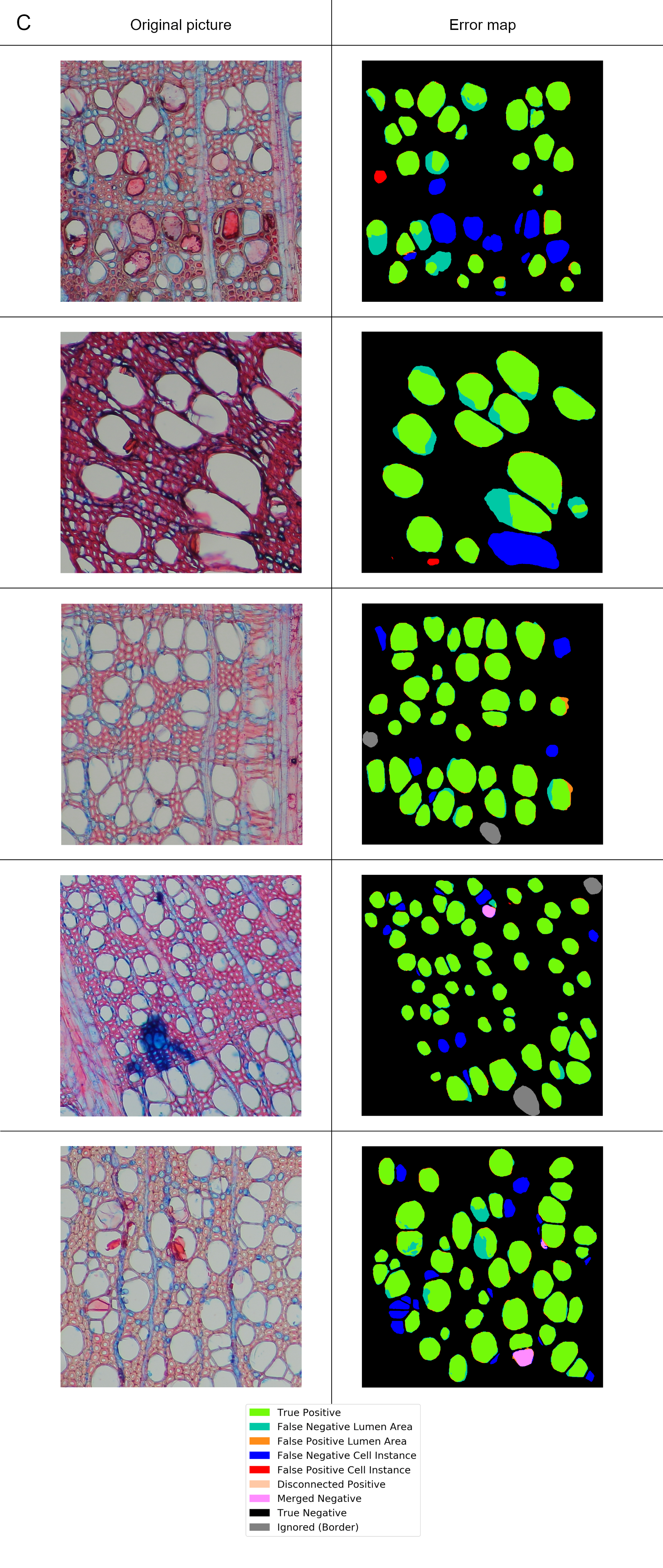


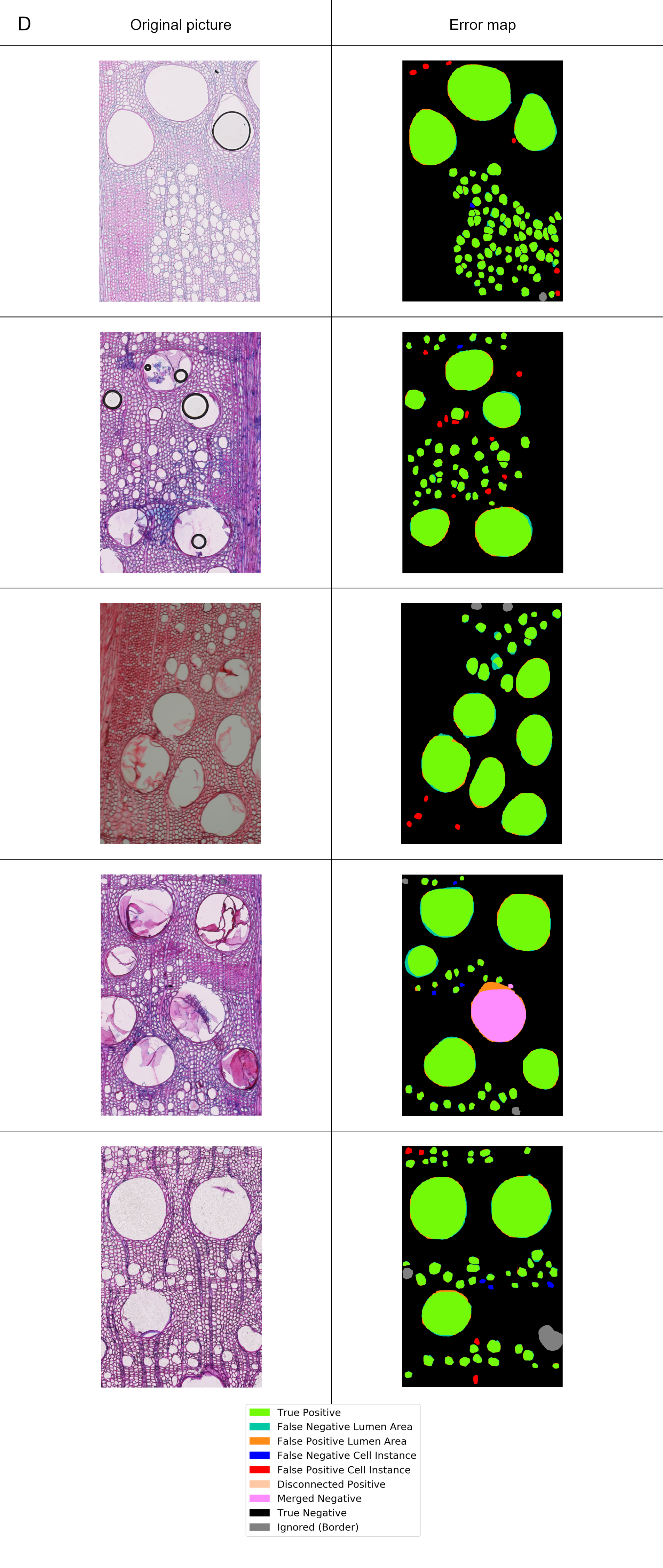


Figure S1. Original pictures and related error map for the sub-dataset of conifers (A), alder (B), beech (C), and oak (D).

Publication bibliography

Lange, Jelena; Carrer, Marco; Pisaric, Michael F. J.; Porter, Trevor J.; Seo, Jeong-Wook; Trouillier, Mario; Wilmking, Martin (2020): Moisture-driven shift in the climate sensitivity of white spruce xylem anatomical traits is coupled to large-scale oscillation patterns across northern treeline in northwest North America. In *Global change biology* 26 (3), pp. 1842–1856. DOI: 10.1111/gcb.14947.

Pampuch, Timo; Anadon-Rosell, Alba; Zacharias, Melanie; Arx, Georg von; Wilmking, Martin (2020): Xylem Anatomical Variability in White Spruce at Treeline Is Largely Driven by Spatial Clustering. In *Frontiers in plant science* 11, p. 581378. DOI: 10.3389/fpls.2020.581378.
